# The correlation of background parenchymal enhancement in the contralateral breast with patient and tumor characteristics of MRI-screen detected breast cancers

**DOI:** 10.1371/journal.pone.0191399

**Published:** 2018-01-19

**Authors:** Suzan Vreemann, Albert Gubern-Mérida, Cristina Borelli, Peter Bult, Nico Karssemeijer, Ritse M. Mann

**Affiliations:** 1 Department of Radiology and Nuclear Medicine, Radboud University Medical Center, Geert Grooteplein 10, Nijmegen, the Netherlands; 2 Department of Radiology, Casa Sollievo della Sofferenza, San Giovanni Rotondo Foggia, Italy; 3 Department of Pathology, Radboud University Medical Center, Nijmegen, the Netherlands; University of Nebraska Medical Center, UNITED STATES

## Abstract

**Purpose:**

Higher background parenchymal enhancement (BPE) could be used for stratification of MRI screening programs since it might be related to a higher breast cancer risk. Therefore, the purpose of this study is to correlate BPE to patient and tumor characteristics in women with unilateral MRI-screen detected breast cancer who participated in an intermediate and high risk screening program. As BPE in the affected breast may be difficult to discern from enhancing cancer, we assumed that BPE in the contralateral breast is a representative measure for BPE in women with unilateral breast cancer.

**Materials and methods:**

This retrospective study was approved by our local institutional board and a waiver for consent was granted. MR-examinations of women with unilateral breast cancers screen-detected on breast MRI were evaluated by two readers. BPE in the contralateral breast was rated according to BI-RADS. Univariate analyses were performed to study associations. Observer variability was computed.

**Results:**

Analysis included 77 breast cancers in 76 patients (age: 48±9.8 years), including 62 invasive and 15 pure ductal carcinoma in-situ cases. A negative association between BPE and tumor grade (p≤0.016) and a positive association with progesterone status (p≤0.021) was found. The correlation was stronger when only considering invasive disease. Inter-reader agreement was substantial.

**Conclusion:**

Lower BPE in the contralateral breast in women with unilateral breast cancer might be associated to higher tumor grade and progesterone receptor negativity. Great care should be taken using BPE for stratification of patients to tailored screening programs.

## Introduction

In dynamic contrast enhanced breast magnetic resonance imaging (DCE-MRI), normal breast parenchyma may enhance after administration of a contrast agent. This enhancement is known as background parenchymal enhancement (BPE). The level of BPE after contrast administration is highly variable between women. Multiple factors, including age, pre- or postmenopausal status, phase in the menstrual cycle, and hormone usage can affect glandular tissue enhancement. Younger women have more often higher BPE and the degree of BPE naturally decreases with age [1].

Previous research showed that BPE may obscure or mimic lesion enhancement and can decrease the accuracy of breast MRI [2–6], even though contradictory results exist for its effect on sensitivity [4, 7]. Nonetheless, lesion demarcation is impaired and it was shown that high BPE increases the likelihood of positive resection margins [8]. According to the newest BI-RADS MRI-lexicon, BPE can be qualitatively evaluated rating the degree of enhancement as minimal, mild, moderate, or marked [9, 10].

Recent literature focused more on the relationship between BPE and breast cancer occurrence. A study examining the relationship between breast cancer and BPE concluded that higher BPE is associated with a higher likelihood of breast cancer development [11]. Odds ratios increased from minimal to marked BPE by a factor of three to ten. This may have strong implications for personalized screening strategies, as these may be adapted to the level of BPE observed. This would be similar to using breast density in mammography as a parameter for stratification of women into more personalized screening programs. This is of particular interest in women at a lifetime risk of 20 to 50%, because cost-effectiveness of MRI screening in these women is often doubted [12, 13].

This raises the question whether high BPE is associated to the occurrence of all breast cancers or just a specific subset of cancer types, as this may affect the usefulness of BPE as a tool for stratification of women to more tailored screening programs. Since tumor size, tumor grade, hormone receptor status, and nodal status are the most essential parameters for long-term outcome prediction [14], it is essential to understand the correlation between BPE and these factors. However, only a few studies investigate the relationship between BPE in breast cancer patients and prognostic factors [15, 16], and none evaluate cancers detected only through screening.

Therefore, the purpose of this study is to correlate BPE to patient and tumor characteristics in women with unilateral breast cancer detected by a screening MRI examination who participated in an intermediate and high risk screening program. As BPE in the affected breast may be difficult to discern from enhancing cancer, we assumed that BPE in the contralateral breast is a representative measure for BPE in women with unilateral breast cancer.

## Materials and methods

### Screening program

This retrospective study was approved by our local institutional review board (CMO Arnhem-Nijmegen) and the requirement for informed consent was waived. The breast cancer screening program for women at increased breast cancer risk (≥ 20–25% lifetime risk) at our institution consists of annual breast MRI in women aged from 25 to 60 in *BRCA* mutation carriers. In women of 30 years or older MRI is combined with mammography. In women at high familial risk, screening starts at 35 or 45 years combining MRI and mammography [17]. The examinations are generally acquired on the same day, although some women prefer to undergo mammography and breast MRI sequentially at six month intervals.

### Case selection

The local database of all breast MR imaging records was searched to identify all screening MR examinations performed between January 2003 and January 2014. Imaging data were cross-referenced with pathology records to identify all malignant lesions in this population. The inclusion criterion was histopathologically proven screen-detected breast cancer (invasive cancer or pure ductal carcinoma in situ (DCIS)). Screen-detected cancers were defined as cancers diagnosed after diagnostic workup initiated by screening findings. Women with a personal history of breast cancer, women who received radiation to the chest at young age, and women who received hormone replacement therapy were excluded. Pathology records were reviewed to determine tumor characteristics according to the Dutch Guidelines for breast cancer [17]. Tumor characteristics were: histological type, histological grade (according to the modified Elston and Ellis criteria [14]), hormone receptor status (using immunohistochemistry for the estrogen receptor (ER) and progesterone receptor (PR) status and fluorescence in situ hybridization (FISH) for the human epidermal growth factor receptor 2 (HER2) status [18, 19]), molecular subtype, primary tumor size (pT-stage) and lymph node status (pN-stage). Molecular subtype was defined based upon receptor status and proliferation markers, as described previously in more detail [20]. One year of follow-up was available for all patients.

### Image acquisition

Breast DCE-MRI acquisitions were performed on either a 1.5 or 3 Tesla Siemens scanner (Magnetom Avanto, Magnetom Sonata, Magnetom Simphony or Magnetom Trio). All women were scanned in prone position using a dedicated bilateral breast coil. A transverse or coronal three-dimensional T1-weighted gradient-echo (GRE) dynamic sequence was performed before contrast agent administration followed by 4 or 5 post-contrast sequences. Subtraction series were created for all post-contrast time points. Motion correction was applied [21]. Pixel spacing (from 0.664 mm to 1.5 mm), slice thickness (from 1 mm to 1.5 mm), matrix (256 x 128, 448 x 381 or 512 x 96 pixels), echo time (from 1.71 msec to 4.76 msec), repetition time (from 4.56 msec to 8.41 msec) and flip angle (from 10° to 25°) differed among acquisitions because of the long time span of this study and the use of various scanners and protocols. Gadolinium based contrast agents were administered at doses of 0.1 mmol/kg or 0.2 mmol/kg using a power injector (Medrad, Warrendale, PA) at a flow rate of 2.5 ml/s, followed by a saline flush. Premenopausal women were scheduled between the sixth and twelfth day of their menstrual cycle.

### MR interpretation

All MR examinations of women with histopathologically proven unilateral breast cancer were reviewed by two experienced readers (a fifth year resident with experience in breast imaging (C.B.) and an experienced radiologist with nine years’ experience in breast MR imaging (R.M.M.)). The readers were informed of the location of the cancer but they were blinded to all other information. The two readers independently evaluated both the level of BPE in the contralateral breast and the level of motion for the complete volume, since motion might result in subtraction artefacts that might be mistaken for BPE. BPE was visually assessed according to the BI-RADS MRI-lexicon as minimal, mild, moderate, or marked on the first post-contrast subtraction series obtained at approximately 90 seconds after contrast administration [10]. Motion was rated likewise on the same volumes as minimal, mild, moderate or severe.

### Statistical analyses

We performed univariate analysis (Chi-square tests for categorical variables and Student’s T-test and one-way ANOVA for continuous variables) to investigate whether BPE was associated to certain patient and tumor characteristics (age, menopausal state, invasive versus in-situ disease, cancer type, tumor grade, ER status, PR status, HER2 status, molecular subtype, size of the primary tumor (pT-stage) and nodal status (pN-stage)). In addition, we investigated the relation between BPE and scored motion in the same manner. To assess inter-reader variability, linear weighted kappa statistics (к) were calculated. The strength of the kappa agreement was defined as <0.000 = poor, 0.000–0.200 = slight, 0.201–0.400 = fair, 0.401–0.600 = moderate, 0.601–0.800 = substantial and 0.801–1.000 = almost perfect.

Because the difference between minimal and mild, and moderate and marked is in clinical practice difficult to make, we chose to dichotomize BPE values in a second step. BPE was dichotomized into low BPE (original scores: minimal and mild) and high BPE (original scores: moderate and marked) to obtain more stable results. Separate analyses were performed for all included cancers and for invasive cancers only separately, and for cancers detected in *BRCA* patients and non-*BRCA* patients. A two-sided p-value of ≤ 0.05 was considered statistically significant. All statistics were performed in SPSS (version 22, SPSS Inc., Chicago, IL).

## Results

In the period from January 2003 to January 2014, 10122 screening MR scans were performed in 2798 women. The cohort consisted of *BRCA* mutation carriers, women with a (strong) family history of breast cancer, women with a personal history of breast cancer, and women with other reasons for inclusion (including women with a germ line PTEN mutation, women who had previous radiation to the chest at young age, and women who were diagnosed with lobular carcinoma in-situ in a previous biopsy). In total, 92 breast cancers (in 91 women) were screen-detected. Fifteen cancers in twelve women were excluded since these women had a personal history of breast cancer (N = 12), radiation to the chest (N = 2) or received hormone replacement therapy (N = 1). Final analysis included 77 cancers in 76 patients (median age of 48 years, range: 24–76 years). One woman had two primary breast cancers in the same breast (an invasive lobular cancer and an invasive ductal cancer).

### Cancers

**Fig 1:**
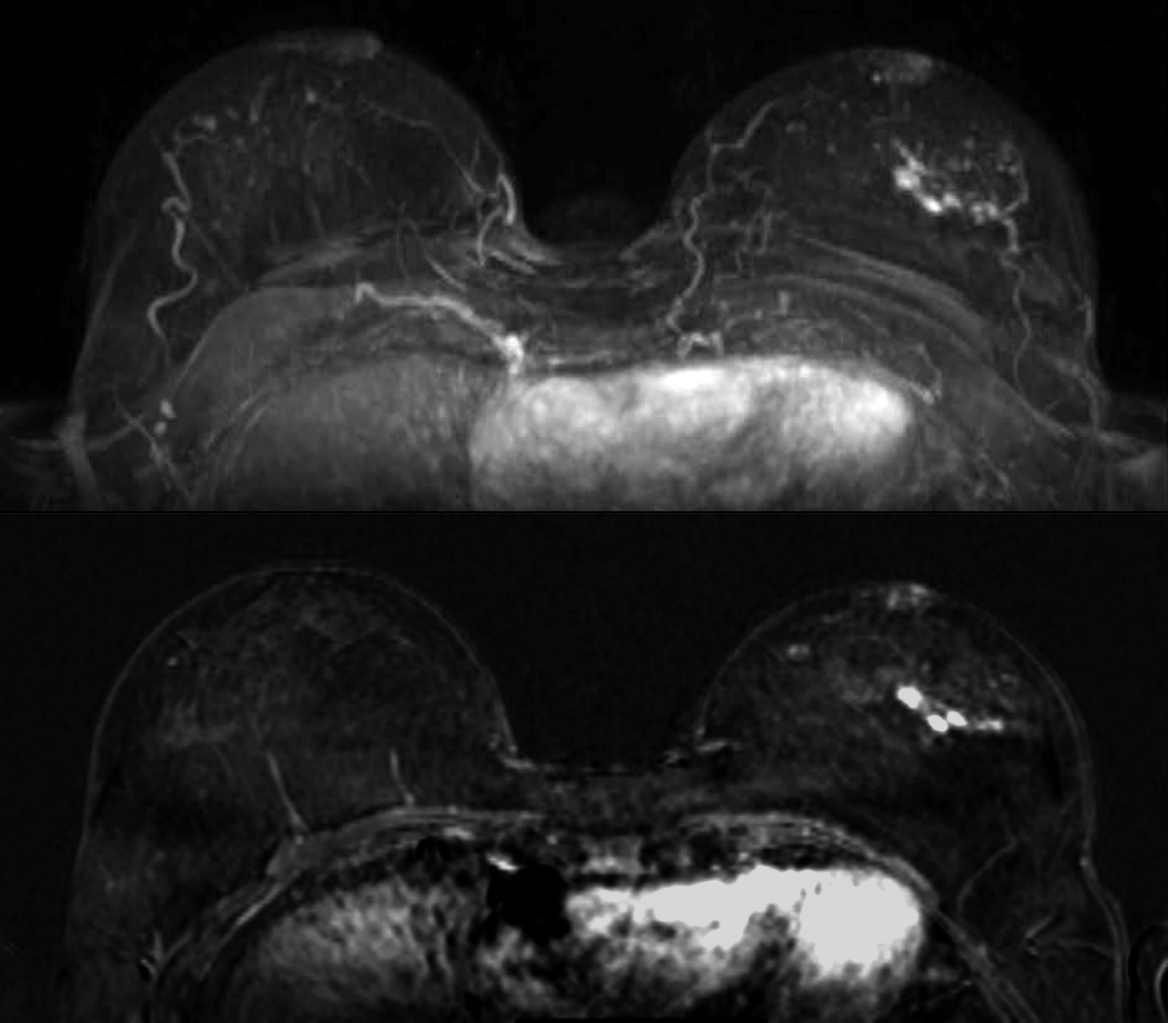
Maximum Intensity Projection (MIP, top image) and subtraction image (bottom image) of woman with unilateral cancer (59 years old, breast tumor in the left breast, IDC grade 2) with BPE rated as minimal by both readers.

**Fig 2:**
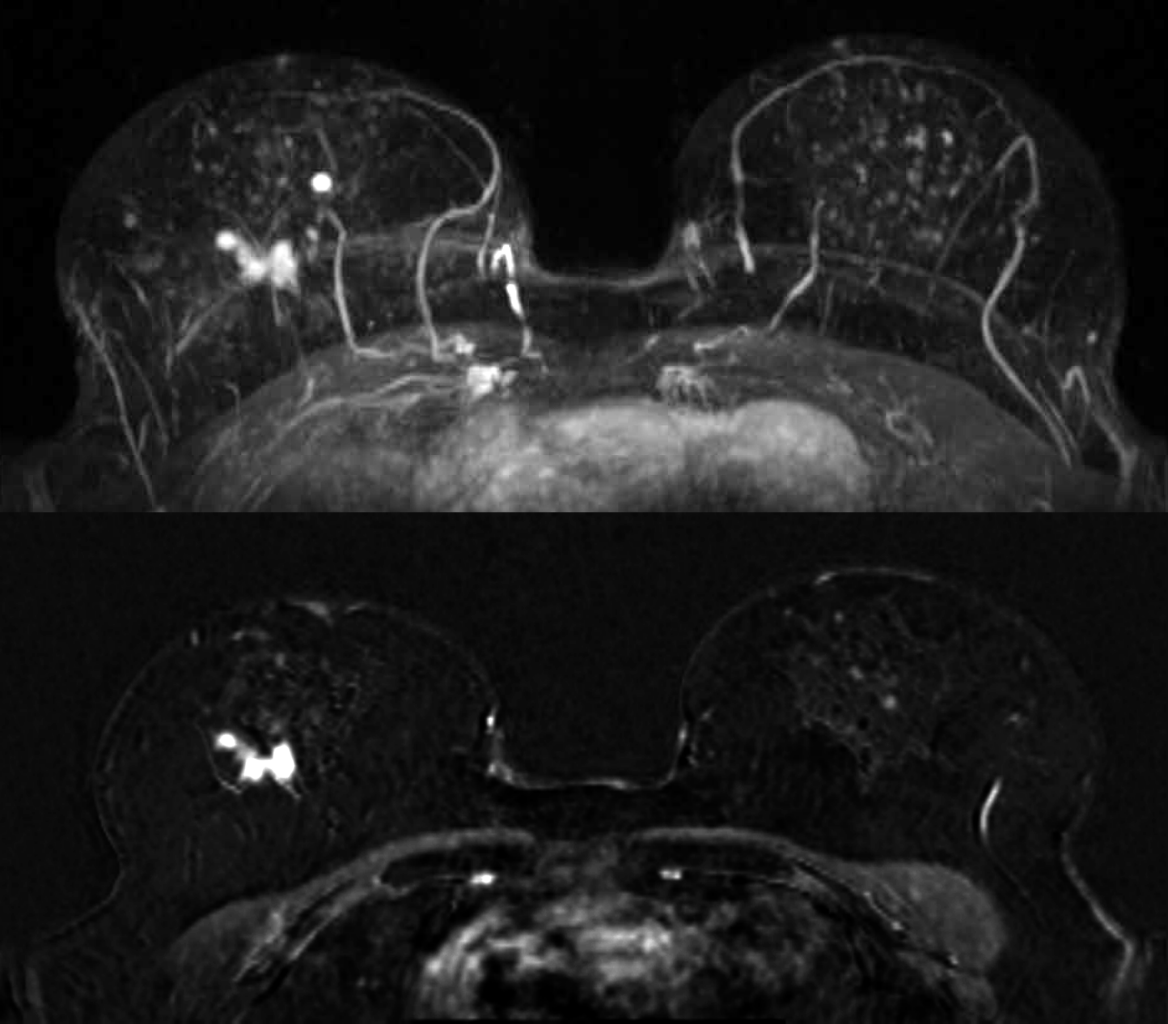
Maximum Intensity Projection (MIP, top image) and subtraction image (bottom image) of woman with unilateral cancer (61 years old, breast tumor in the right breast, DCIS grade 2) with BPE rated as mild by both readers.

**Fig 3:**
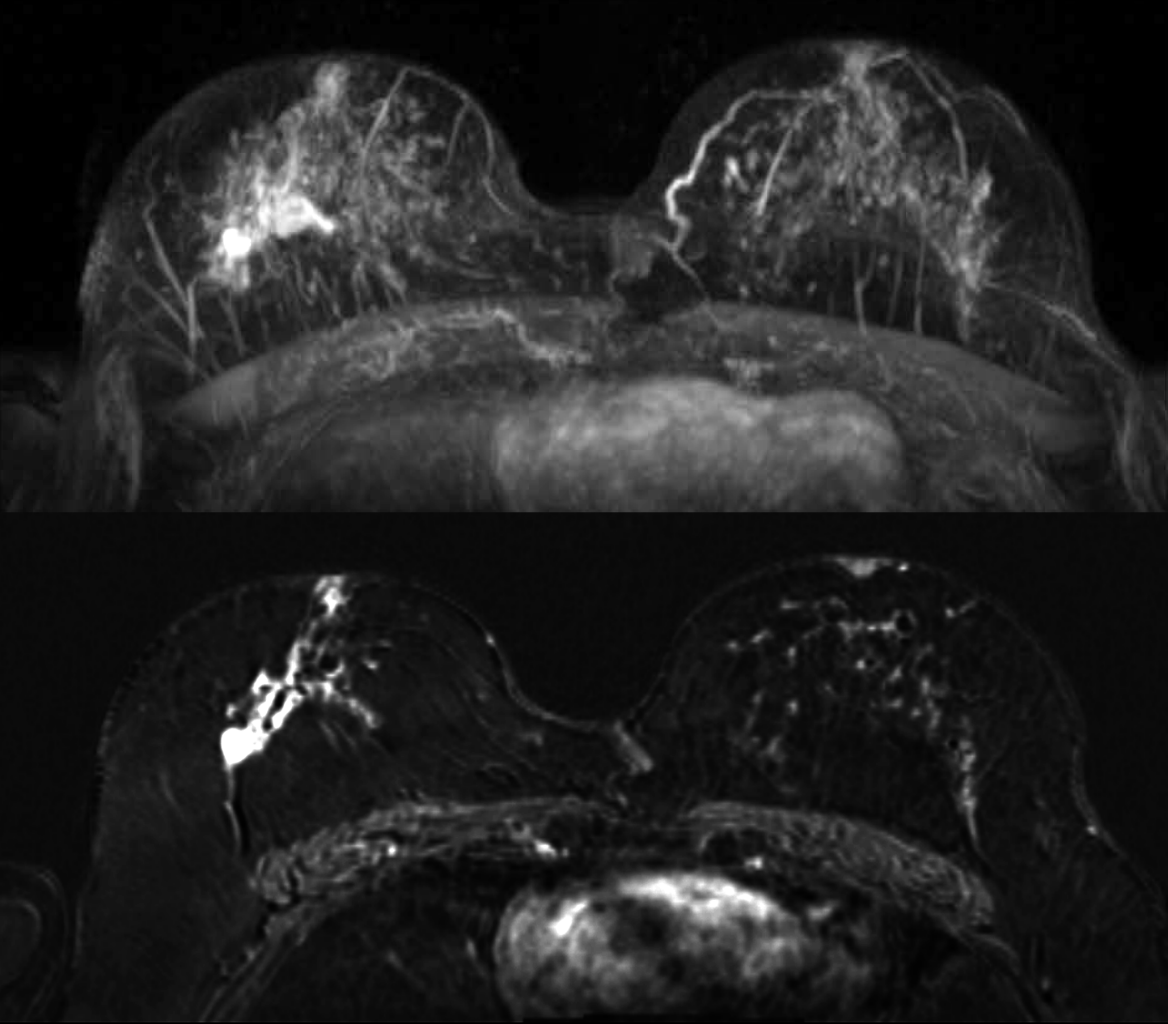
Maximum Intensity Projection (MIP, top image) and subtraction image (bottom image) of woman with unilateral cancer (70 years old, breast tumor in the right breast, IDC grade 2) with BPE rated as moderate by both readers.

**Fig 4:**
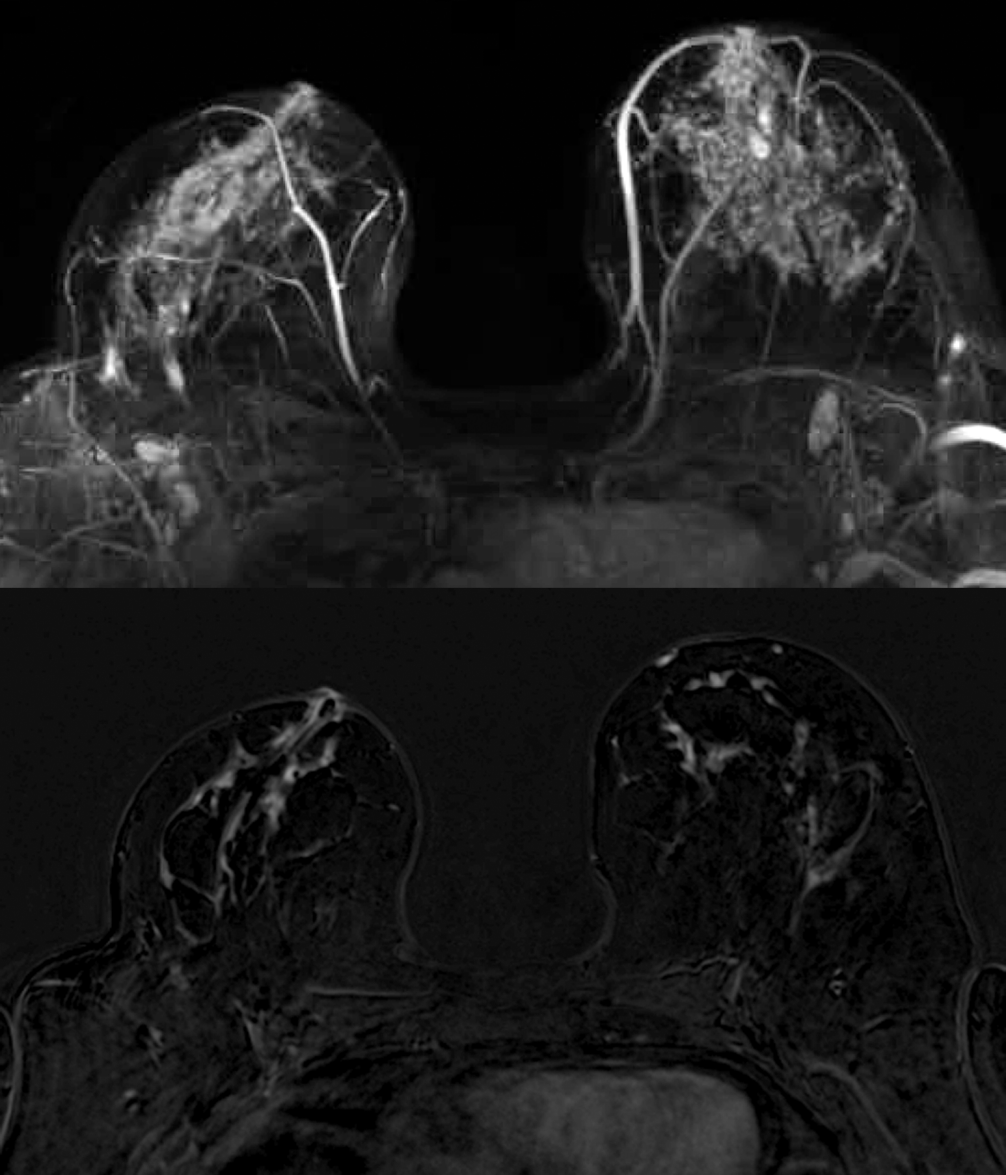
Maximum Intensity Projection (MIP, top image) and subtraction image (bottom image) of woman with unilateral cancer (60 years old, breast tumor in the left breast, IDC grade 1) with BPE rated as marked by both readers.

Patient and tumor characteristics are given in Table 1. The significant results of univariate analysis of patient and tumor characteristics in relation to BPE are presented in Table 2. BPE seems to be associated to tumor grade, scored motion, and to the PR status of the cancer. A substantial agreement in the assessment of BPE was found between R1 and R2 for all the cancers (к = 0.719 (95% CI: 0.615–0.824)) and for invasive cancers only (к = 0.750 (95% CI: 0.640–0.861)) using the original 4 categories. When using 2 categories the agreement was still substantial (к = 0.633 (95% CI: 0.449–0.819)) for all cancers and for invasive cancers only (к = 0.677 (95% CI: 0.488–0.865)) Figs 1–4 show examples of the four BPE categories.

**Table 1:** Patient and cancer characteristics.

Age	48 years ± 9.9 years (range: 24–76 years)
**Menopausal state**	N (%)
Premenopausal	36 (47%)
Postmenopausal	41 (53%)
**Cancer types**	N (%)
*DCIS*	15 (19%)
Grade 1	0 (0%)
Grade 2	9 (60%)
Grade 3	6 (40%)
*Invasive*	62 (81%)
IDC	51 (82%)
ILC	10 (16%)
Other	1 (2%)
Grade 1	11 (18%)
Grade 2	22 (36%)
Grade 3	29 (47%)
ER status	N (%)
Positive	41 (66%)
Negative	20 (32%)
Unknown	1 (2%)
PR status	N (%)
Positive	30 (48%)
Negative	31 (50%)
Unknown	1 (2%)
HER2 status	N (%)
Positive	10 (16%)
Negative	50 (81%)
Unknown	2 (3%)
Molecular subtypes	N (%)
Luminal A	37 (60%)
Luminal B	3 (5%)
HER2 type	7 (11%)
Basal-like	13 (21%)
Unknown	2 (3%)
T-stage (invasive only)	N (%)
Stage 1	42 (68%)
Stage 2	17 (27%)
Stage 3	1 (2%)
Unknown	2 (3%)
N-stage (invasive only)	N (%)
Stage 0	38 (61%)
Stage 1	20 (32%)
Stage 2	1 (2%)
Stage 3	1 (2%)
Unknown	2 (3%)

DCIS: Ductal carcinoma in-situ; IDC: Invasive ductal carcinoma; ILC: Invasive lobular carcinoma; ER: Estrogen receptor; PR: Progesterone receptor; HER2: Human epidermal growth factor receptor 2

**Table 2:** Results of univariate analysis for BPE.

Investigated parameters in association with BPE	Original scores BPE by R1	Original scores BPE by R2	P-value R1^$^	P-value R1^#^	P-value R2^$^	P-value R2^#^
*Grade*	grade 1/ 2 /3	grade 1/ 2 /3	0.040*	0.016*	0.008*	0.003*
1. Minimal BPE	1/14/14	0/13/14				
2. Mild BPE	2/7/12	4/10/17				
3. Moderate BPE	5/5/8	5/5/3				
4. Marked BPE	3/5/1	2/3/1				
Missing: 0						
*PR status*	PR+/ PR-	PR+/ PR-	0.089	0.018*	0.015*	0.021*
1. Minimal BPE	7/14	5/16				
2. Mild BPE	7/10	13/11				
3. Moderate BPE	11/5	8/3				
4. Marked BPE	5/2	4/1				
Missing: 17						
*Scored motion per reader*	minimal/ mild/ moderate/ and severe	minimal/ mild/ moderate/ and severe	0.038*	0.009*	0.031*	0.066
1. Minimal BPE	17/8/3/1	20/7/0/0				
2. Mild BPE	17/3/1/0	19/6/6/0				
3. Moderate BPE	4/7/5/2	7/6/0/0				
4. Marked BPE	4/3/2/0	4/2/0/0				
Missing: 0						

^$^ P-values are based on original data (including the 4 BPE categories) using Chi-square tests

^#^ P-values are based on dichotomized values using Chi-square tests

* is indicating a p-value ≤ 0.05.

PR: Progesterone receptor. Insignificant factors included: age (p≥0.284 for original BPE values and p≥0.188 for dichotomized BPE values), menopause (p≥0.119and p≥0.055), invasiveness (p≥0.383and p≥0.234), cancer type (p≥0.284 and p≥0.055), T-stage (p≥0.242 and p≥0.230), N-stage (p≥0.356 and p≥0.175), molecular subtype (p≥0.165 and p≥0.061), ER-status (p≥0.119 and p≥0.055), and HER2-status (p≥0.700 and p = 0.999).

### Associations

**Fig 5:**
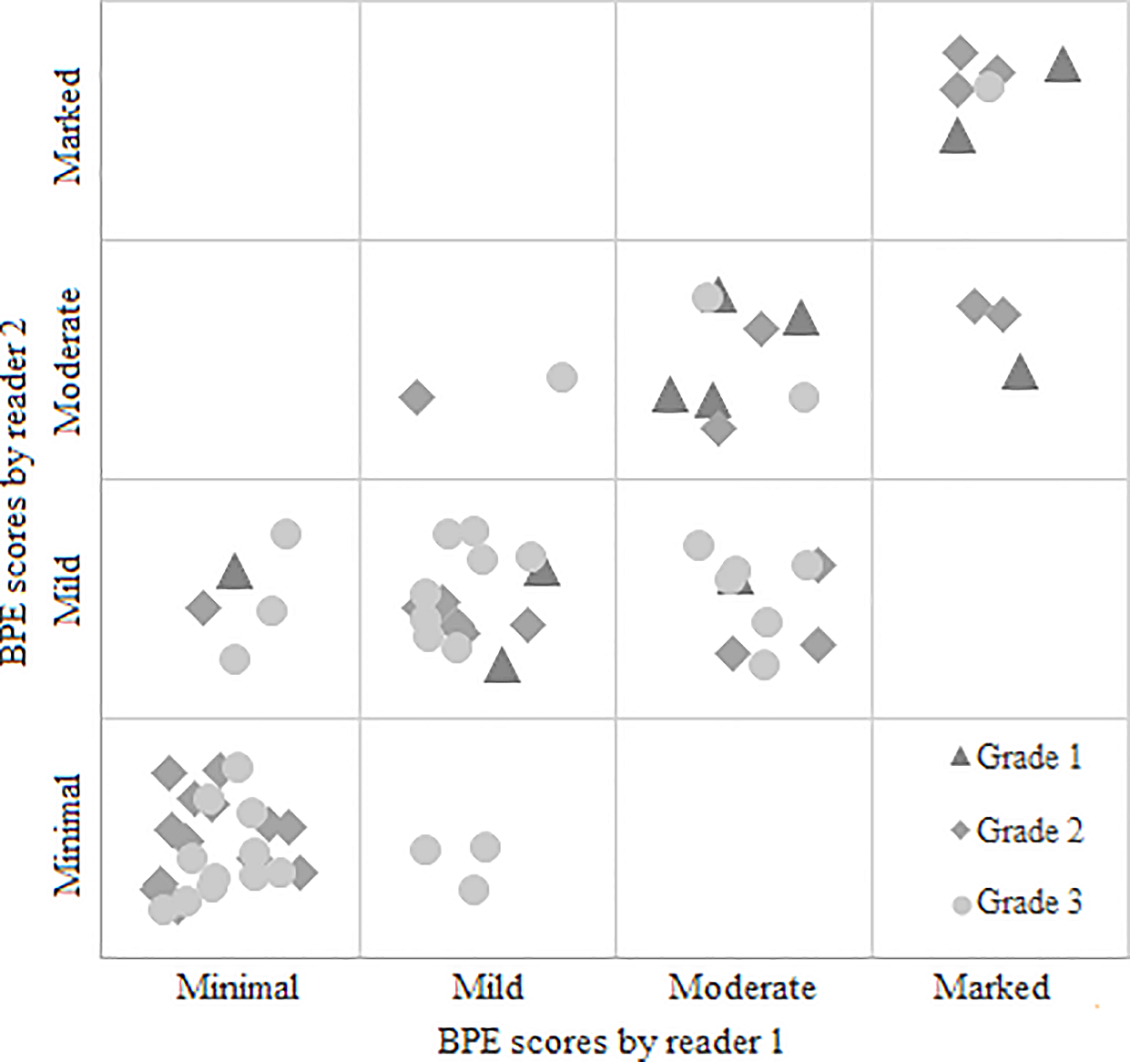
The association between BPE and tumor grade for Reader 1 and Reader 2.

For both readers there was a significant negative association between BPE and pathological tumor grade (R1: p = 0.016 and R2: p = 0.003 for all cancers, R1: p = 0.031 and R2 = 0.007 for invasive cancers only, using dichotomized BPE scores, Fig 5).

We investigated the association in *BRCA* mutation carriers and non-*BRCA* patients separately. The results show that there was no association between BPE and tumor grade in *BRCA* mutation carriers (p = 0.175 for both readers), however, there was a significant negative association in non-*BRCA* patients for reader 2 (p = 0.001).

**Fig 6:**
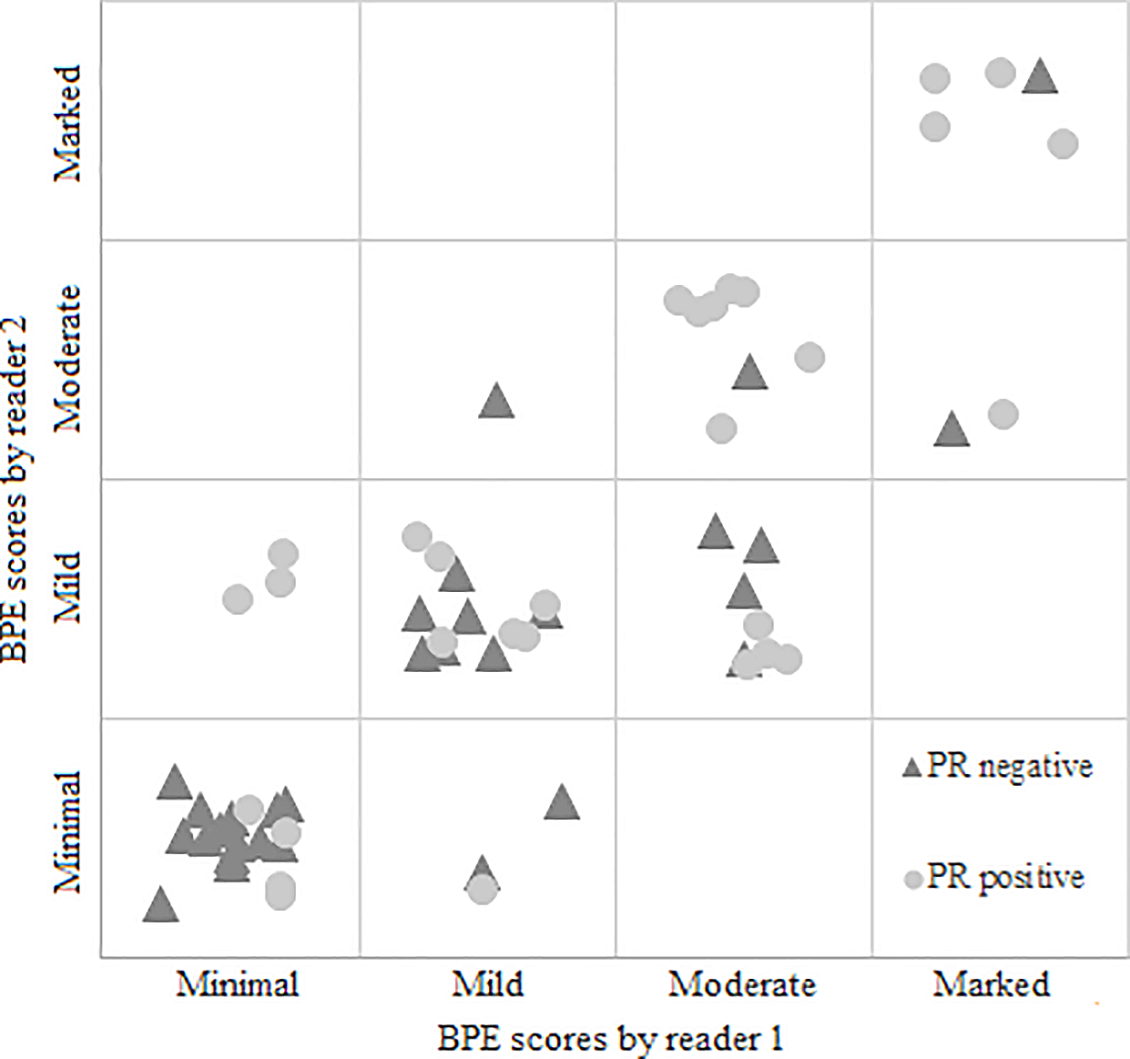
The association between BPE and progesterone status (PR status) for Reader 1 and Reader 2.

We did also observe a significant positive association between BPE and motion in one reader (R1; p = 0.009) and a significant positive association between BPE and PR status for both the readers (R1; p = 0.018 and R2; p = 0.021, Fig 6).

## Discussion

In this study, we investigated the association between BPE and patient and tumor characteristics in women at increased risk with screen-detected unilateral breast cancer. Our results show that there is a negative association between BPE and tumor grade. Furthermore, a positive association between BPE and PR status is observed.

There is a worldwide intention to shift current research of breast cancer screening from population based screening towards personalized screening. Based upon the results of King et al. [11] and the study of Telegrafo et al. [22], who detected a strong positive correlation between BPE and breast cancer risk, BPE has the potential to be used as a stratification factor for personalized breast screening, although this was not evident in all studies [4, 23]. Considering the negative association between BPE and grade in our study, this would however mean that women who tend to develop low grade tumors might be more intensively screened than women who tend to develop high grade tumors. Similarly, the possible association between BPE and PR status should be taken into account.

The association between BPE and grade might be biologically explained by the fact that low grade cancers are in general also hormone receptor positive, whereas more aggressive cancers may be hormone receptor negative [24]. This might also explain the positive association observed between tumor PR status and BPE. It has been shown that increased estrogen and progesterone levels are related to an increased BPE [25, 26], but also to increased breast cancer risk [27–32], especially for hormone receptor positive cancers [33], consequently relating high BPE to hormone receptor positive cancers. However, we did not observe a correlation between ER status and BPE for any of the readers.

Our study also hints at a different pathogenesis for high grade tumors, in which hormonal stimulation is less important. This supports the theory that breast cancer development for high grade tumors is vastly different from low grade tumors [34, 35]. This can also explain the difference between *BRCA* and non-*BRCA* patients (frequently sporadic tumors), as hormonal stimulation pathways might be different in *BRCA* tumor development, as described for the *BRCA1* gene by Hu et al. [36]. Our results may also partly explain the findings of van der Velden et al. [15]. The authors showed that parenchymal enhancement in the contralateral breast of women with invasive breast cancer is correlated to long-term outcome. Lower values of parenchymal enhancement showed potential as a predictive biomarker for relatively poor outcome in women who received endocrine therapy. This might be due to differences in tumor grade, but it could also be explained by the lower importance of the hormonal stimulation pathway for breast cancer growth in women with relatively low contralateral BPE. However, it must be taken into account that the definition of parenchymal enhancement of van der Velden et al. is different from the definition described in the BI-RADS MRI-lexicon. In their study, automatically calculated late enhancement of the parenchyma (percentage of parenchymal enhancement over the whole post-contrast period) was used.

We found an association between BPE and increased motion scores in one reader (R1), which may be explained by the fact that motion can be misleading and might be incorrectly considered as BPE in subtraction images and vice versa [37, 38]. However, further research is needed to investigate whether this holds true in a larger dataset and with more experienced readers.

Our findings are in contrast to the, to our knowledge, only study that so far investigated the relation between BPE and tumor characteristics [16]. Kim et al. reported that BPE of the contralateral breast is independent of tumor characteristics. Likely, this difference can be explained by the differences in patient cohorts. We only included women participating in an intermediate and high risk screening program, whereas Kim et al. [16] included all patients with invasive ductal cancers who underwent preoperative MRI. The patients in our study had a different risk profile, although age (age under 50 years: 53.8% in current study versus 51.7% in the study of Kim et al.) and menopausal status (pre-menopausal: 45.0% in current study versus 47.9% in the study of Kim et al.) between groups were comparable. We chose to study this population because these women are regularly screened using MRI and might benefit from personalized screening programs.

We calculated Chi-square tests to study associations between BPE and tumor characteristics. This was chosen over performing an analysis calculating odds ratios (including more variables as age and menopausal state) because tumor grade was the only prognostic factor that showed a significant relation to BPE in both of the readers, and the limited number of cases.

Our study has some limitations. Despite the fact that the number of cancers reported on in a high-risk screening program is relatively large, the absolute number of cancers included to study the correlations is relatively low. This holds particularly true for the number of pure DCIS cases (n = 15), although 19% pure DCIS (15/77) is a realistic representation of screening practice [39]. Furthermore, we suspect that the relatively low number of cancers might be one of the main reasons for not finding a significant association between BPE and age. Other studies showed a strong negative correlation of age with BPE [4, 25]. This small sample size is also the main reason why no multivariable statistical methods are used in the current study. Future research is needed to test for possible confounders and interaction terms. In addition, continuous values of BPE could be used in future research to be able to define clear cut-offs.

Lastly, because of the long time span of this study, there is a large variability of MRI acquisitions in this dataset. Different MRI field strengths (1.5T and 3T), breast coils (4-channel, 7-channel and 16-channel) and MRI protocols were used, and different types and amounts of contrast agents were injected. This may have strongly affected the amount of enhancement seen in the scans. However, the rating of BPE according to the BI-RADS MRI-lexicon only considers the visual assessment of the fraction of fibroglandular tissue that enhances at 90 seconds after contrast administration and does not change based on differences in peak enhancement or wash-out pattern, which are more likely to be affected by the variability in scanning parameters. To our knowledge, only the study of Uematsu et al. [40] directly compared BPE in breast cancer patients at 1.5T and 3T and found no differences in the assessment between field strengths. The large variability, on the other hand, has the advantage that it reflects many of the breast MRI protocols currently in use and our findings therefore seem extendable to breast MRI screening in general.

In conclusion, BPE in the contralateral breast of patients with unilateral breast cancer in an intermediate and high-risk population might be negatively related to tumor grade and positively related to progesterone receptor status. Based on this finding, great care must be taken before using BPE as a method to stratify women at increased risk to more personalized MRI screening strategies. These results should, however, be validated in a larger study.

## Supporting information

S1 File SPSS table of the raw study data. (SAV)
